# Relationship Between Subjective Reports of Temporary Threshold Shift and the Prevalence of Hearing Problems in Military Personnel

**DOI:** 10.1177/2331216519872601

**Published:** 2019-09-16

**Authors:** Douglas S. Brungart, Mary E. Barrett, Jaclyn Schurman, Benjamin Sheffield, Leilani Ramos, Roberta Martorana, Hector Galloza

**Affiliations:** 1 Walter Reed National Military Medical Center, Bethesda, MD, USA; 2 Oak Ridge Institute for Science and Education (ORISE), Oak Ridge, TN, USA; 3 Henry Jackson Foundation, Bethesda, MD, USA; 4 Army Public Health Center, Aberdeen Proving Ground, MD; 5 KE'AKI Technologies, LLC, Frederick, MD; 6 2GARI Solutions Inc., Aguada, PR

**Keywords:** noise exposure, synaptopathy, temporary threshold shift

## Abstract

Traditional hearing conservation programs are based on the premise that noise dose, as measured by the time-weighted average noise level, is the primary risk factor associated with occupational hearing loss and that permanent threshold shifts are the most relevant outcome measures for determining when a noise-related hearing loss has occurred. However, recent studies in animal models have suggested that significant neurological damage to the hearing system can occur from noise exposures even when they are not severe enough to result in permanent threshold shifts. This has led to a number of studies attempting to relate subjective measures of noise exposure to subjective measures of hearing difficulty and suprathreshold measures of hearing performance (e.g., speech-in-noise tests). In this study, 3,330 U.S. service members volunteered to complete a survey on noise exposure, subjective hearing complaints, and tinnitus in conjunction with their annual hearing tests. Two questions were also included about the frequency and duration of temporary hearing losses that may have been experienced by the service member. The results show that subjective reports of temporary threshold shifts were substantially more predictive of tinnitus and other hearing complaints than more traditional questions based on the frequency of noise exposure.

## Introduction

Noise exposure is an occupational risk in many professions, but some of the most hazardous noise exposures occur in military environments. In addition to continuous noise exposures from vehicles and machinery, service members (SMs) may also experience repeated doses of impulsive noise from small and large caliber firearms and occasional doses of very high-level noise or blast overpressure from heavy weapons fire, grenades, demolition explosions, improvised explosive devices, or incoming enemy fire.

Traditional hearing conservation programs are based on the assumption that the primary risk associated with noise exposure is sensorineural hearing loss, which manifests as a shift in the intensity level of the quietest pure tone a listener can detect.

The Occupational Safety and Health Administration (OSHA) and the National Institute for Occupational Safety and Health (NIOSH) rules require noise-exposed individuals to receive periodic hearing tests to ensure that no shifts in pure-tone threshold have occurred, and these rules presume that no noise-related hearing loss has occurred until an individual experiences a standard threshold shift (STS) (NIOSH, 1998; OSHA, 1983). An STS is defined by OSHA as an average increase of 10 dB or more at 2000, 3000, and 4000 Hz in one or both ears, and by NIOSH as an average increase of 15 dB or more at 500, 1000, 2000, 3000, 4000, or 6000 Hz in one or both ears.

Notably, the focus of virtually all current noise monitoring programs is to detect *permanent* threshold shifts (PTSs). Most individuals who are exposed to the maximum allowable dose of noise under OSHA guidelines (90 dB time-weighted average for 8 hours, with a 50% reduction in allowable exposure for each 5 dB increase in noise level above 90 dB; OSHA, 1983) or under NIOSH guidelines (85 dB time-weighted average for 8 hours, with a 50% reduction in allowable exposure time for each 3 dB increase in level above 85 dB; NIOSH, 1998) will experience some temporary increase in hearing thresholds. Although the amount of temporary increase varies from individual to individual, some models have predicted a typical person will begin to experience a temporary threshold shift (TTS) when exposed to continuous noise that exceeds 80 dB-A (Miller, 1974). However, these TTSs have generally been considered non-hazardous as long as the hearing thresholds eventually recover to their pre-exposure baseline. This is why OSHA rules require individuals to wait 14 hours after noise exposure before receiving a hearing test, and NIOSH rules require individuals to wait 12 hours. They also require individuals who register an STS on a hearing test to receive an additional follow up test at least 24 hours later to verify that the observed shift in thresholds is permanent.

In recent years, the assumption that noise-related hearing damage is limited to cases where patients experience a PTS has been called into question. Results from animal models have shown that substantial neurological damage can occur to the cochlear nerve and associated structures even when there is little or no change in pure-tone threshold (Kujawa & Liberman, 2009). Some studies have found that normal hearing individuals who have a history of recreational noise exposure do not experience significant changes in auditory performance, such as on speech-in-noise tasks (Grinn, Wiseman, Baker, & Le Prell, 2017; Grose, Buss, & Hall, 2017). However, some studies have shown that individuals with loud noise exposure may experience hearing problems that are not apparent from their audiometric thresholds, such as individuals with blast exposure (Gallun et al., 2012; Saunders et al., 2015).

Unfortunately, the ability to evaluate the impact of noise exposure on hearing performance is limited. In the ideal case, every person assigned to a hearing conservation program would be provided with a dosimeter that would provide a comprehensive measure of noise exposure for that individual. However, in reality, both hearing program managers and hearing researchers are often limited to the information obtained from subjective instruments that attempt to assess how much noise individuals were exposed to over a set period of time or, in some cases, over their entire lifetime. For example, the Noise Exposure Questionnaire, or NEQ, is a task-based survey instrument designed to estimate an individual's annual noise dose by estimating the amount of time spent in a set of common noisy tasks (e.g., the use of power tools, piloting a plane, playing an instrument; Johnson, Cooper, Stamper, & Chertoff, 2017). There is also a 1-minute, three-item version of the NEQ designed to screen those who may be exposed to hazardous noise. The Noise Exposure Structured Interview (NESI) is a more extensive structured interview designed to assess lifetime noise exposure (Guest et al., 2018a). The Lifetime Exposure to Noise and Solvents Questionnaire (LENS-Q) is another comprehensive survey instrument that asks about the frequency and duration of participation in potentially noise-hazardous military, occupational, and recreational activities and makes a quantitative estimate of lifetime noise dose that adjusts for the self-reported use of hearing protection in each activity (Bramhall, Konrad-Martin, McMillan, & Griest, 2017; Gordon et al., 2017).

The goal of NEQ, NESI, and LENS-Q is to use subjective survey tools to estimate, as accurately as possible, the quantitative noise dose that would have been measured if the participant had been equipped with a noise dosimeter over the time period of the study. Although estimation of the quantitative dose is a reasonable goal, there may be significant variability in how different listeners will respond to noise exposure (Davis, Kozel, & Erway, 2003; Sliwinska-Kowalska & Pawelczyk, 2013). An alternative metric that might be useful for assessing the risk of noise exposure is one that asks questions about the perceptual consequences of noise exposure, such as the frequency a listener reports experiencing a change in hearing after a noise exposure and the amount of time those changes took to resolve back to normal hearing. Notably, a set of questions similar to these were included in a preliminary version of the NEQ, but were ultimately rejected because they were not found to correlate with overall noise dose (Johnson et al., 2017). Consequently, those questions were not included in subsequent studies that used the NEQ to examine the effect of noise exposure on hearing performance in listeners with near-normal thresholds.

One study that has asked explicit questions about perceived changes in hearing after a noise exposure is a recent study by Le Prell, Siburt, Lobarinas, Griffiths, and Spankovich (2018). In that study, 74 young adult civilians were asked the yes/no question “Have you ever experienced hearing loss after exposure to a loud sound?” Ten of the 74 participants answered yes to this question, and this response was not found to correlate with threshold sensitivity, Distortion product otoacoustic emission (DPOAE) amplitude, or speech-in-noise scores on the Words-in-Noise test. However, no subjective measures of hearing problems were obtained in this population, and it is possible that this sample size was too small or insufficiently noise-exposed to reveal an underlying relationship between self-perceived temporary hearing changes and long-term hearing function.

In the present study, a large cadre of SMs were asked to complete a series of survey questions on noise exposure, TTS, tinnitus, and subjective hearing difficulties in conjunction with their annual pure-tone hearing tests. The results were used to develop predictive models relating the subjective measures of occupational noise exposure to hearing thresholds and subjective hearing complaints. The next section describes the methods in more detail.

## Methods

The questionnaires were administered as part of a tablet-based survey that was conducted in the hearing conservation clinics of seven military bases. Individuals had their hearing tested, and the results of the hearing test were recorded along with their tablet responses. No other personally identifiable information was retained.

## Questions

### Demographic Questions

- **Age**: Age in years at the time of the survey.
- **Gender**: Male or Female.
- **Native Language**: Participants were asked “Do you consider English to be your native language?” and indicated “Yes” or “No.”
- **Years of Active Duty**: Participants were asked “How many years of active service do you have?” and were given five options: “<1 year,” “1 to 3 years,” “3 to 10 years,” “11 to 20 years,” or “>20 years.”
- **Combat Deployment**: Participants were asked “Which option best describes your military service?” and were given the options “No history of military service,” “Active/prior military service with no wartime deployments to Iraq or Afghanistan,” or “Active/prior military service with at least one wartime deployment to Iraq or Afghanistan.”
- **Mild traumatic brain injury (mTBI) Diagnosis**: Participants were asked “Have you ever been diagnosed with a TBI or concussion?” and were given the options “No,” “Only mild TBIs or concussions,” or “One or more moderate or severe TBIs.” A positive mTBI Diagnosis was counted if the participant selected “Only mild TBIs or concussions.”
- *>***mTBI Diagnosis**: Same as the mTBI Diagnosis question. A positive > mTBI Diagnosis was counted if the participant selected “One or more moderate or severe TBIs.”

### Noise History Questions

- **Continuous Noise**: Participants were asked “How often are you exposed to continuous noise that is so loud you need to raise your voice to be heard by a person standing three feet away?” and were given the options “Never,” “Less than once per year,” “Once per year,” “Several times a year,” “Several times a month,” “Several times a week,” and “Daily.”
- **Small Arms Noise**: Participants were asked “How often are you exposed to noise from small-caliber firearms (up to 12.7 mm)?” and were given the options “Never,” “Less than once per year,” “Once per year,” “Several times a year,” “Several times a month,” “Several times a week,” and “Daily.”
- **Heavy Weapons Noise**: Participants were asked “How often are you exposed to heavy weapon fire (mortars, artillery, recoilless rifle) or explosions?” and were given the options “Never,” “Less than once per year,” “Once per year,” “Several times a year,” “Several times a month,” “Several times a week,” and “Daily.”
- **Hearing Protection**: Participants were asked “How often did you wear hearing protection?” and were given the options “Never,” “Some of the time,” “Most of the time,” or “Always.” This question was asked separately for Continuous Noise, Small Arms Noise, and Heavy Weapon Noise. Because the hearing protection question was meaningless for individuals who indicated they were never exposed to a certain type of noise, a decision was made to analyze only one hearing protection variable which was defined as the maximum hearing protection response across all three noise categories. A very small number (<5%) of participants reported they were never exposed to any type of noise. These subjects were assigned an “N/A” response for the hearing protection question that assigned a numerical value equal to the mean response of all subjects who did have a response, which was numerically roughly halfway between the “Some of the Time” and “Most of the Time” responses.
- **Far Blast Exposure**: Participants were asked two questions about blast exposure: “Have you ever been exposed to an explosive blast in combat?” and “Have you ever been exposed to an explosive blast in training?” The participants were given the options “No,” “Yes, but not close enough to feel the heat or pressure wave,” or “Yes, close enough to feel the heat or pressure wave.” A positive Far Blast Exposure was counted if the participant selected “Yes, but not close enough to feel the heat or pressure wave” for either the combat or training question.
- **Close Blast Exposure**: Same as the Far Blast Exposure questions. A positive Close Blast Exposure was counted if the participant selected “Yes, close enough to feel the heat or pressure wave” for either the combat or training question.
- **TTS Frequency**: Participants were asked “Have you ever experienced a temporary change in your hearing (dullness or a muffled sound) after exposure to a loud noise?” and were given the options “Never,” “Once in my lifetime,” “A few times in my lifetime,” “Several times a year,” “Several times a month,” “Several times a week,” and “Every day.” (Note that the term frequency refers to the number of TTS events, rather than the frequency band where the TTS occurred.)
- **TTS Max Duration**: Participants who responded that they had experienced at least one TTS were asked “What is the longest time it has taken for your hearing to recover back to normal?” and were given the options “A few seconds,” “A few minutes,” “A few hours,” “A few days,” and “It never fully recovered.” These responses were assigned the values 1 to 5 on this scale. Participants who indicated they had never experienced a TTS were automatically assigned a 0 value on this question.

### Subjective Symptoms

- **Perceptual Tinnitus**: Participants were asked “Have you ever experienced tinnitus (a roaring, ringing, or buzzing sensation in your ear) that has lasted more than 2 to 3 minutes?” and were given the options “Never,” “Once in my lifetime,” “A few times in my lifetime,” “Several times a year,” “Several times a month,” “Several times a week,” and “Every Day.” Note that this question was derived from the tinnitus and hearing survey (THS; Henry et al., 2015), and that it measures how frequently the listener experiences auditory illusions, but does not address how bothersome they are. Throughout this article, this variable will be referred to as “perceptual tinnitus”.
- **THS**: This questionnaire consisted of the four hearing questions from the THS (Henry et al., 2015). The participants were asked the following questions: (a) “Over the last week, I couldn't understand what others were saying in noisy or crowded places”; (b) “Over the last week, I couldn't understand what people were saying on TV or in movies”; (c) “Over the last week, I couldn't understand people with soft voices”; and (d) “Over the last week, I couldn't understand what was being said in group conversations.” The participants were asked to select the number that represented where they fell on a range of 0 to 10 (0 = *No, not a problem*; 10 = *Yes, a very big problem*).
- **SSQ Survey**: This questionnaire consisted of six questions derived from the Spatial and Sound Qualities (SSQ) questionnaire (Gatehouse & Noble, 2004). The participants were asked the following questions: (a) “You are talking to someone on the telephone and someone next to you starts talking. Can you follow what is being said by both talkers?”; (b) “You are in a group and the conversation switches from one person to another. Can you easily follow the conversation without missing the start of what each new speaker is saying?”; (c) “You are talking to one person. There is continuous background noise, such as a fan or running water. Can you follow what the person says?”; (d) “Do you have to concentrate very much when listening to someone or something?”; (e) “Do everyday sounds that you hear seem to have an artificial or unnatural quality?”; and (f) “Can you easily ignore other sounds when trying to listen to something?” The participants were asked to select the number that represented where they fell on a range of 0 to 10. For questions 1 to 3, the scale ranged from 0 = *Not at all* to 10 = *Perfectly*. For question 4, the scale ranged from 0 = *Concentrate hard* to 10 = *No need to concentrate*. For question 5, the scale ranged from 0 = *Unnatural* to 10 = *Natural*. For question 6, the scale ranged from 0 = *Not easily ignored* to 10 = *Easily ignored*. Note that these scales are reversed relative to those in the THS (i.e., higher numbers indicate less difficulty rather than more difficulty). For analysis purposes, the SSQ values were subtracted from 10 to make them consistent with the other measures in the study (i.e., so increasing numbers indicated increasing hearing difficulty).
- **Auditory Localization**: Participants were asked “How well are you able to determine the locations of sounds that you hear?” and were asked to select the number that represented where they fell on a range of 0 to 10 (0 = *Not at all*; 10 = *Perfectly*).
- **Noise Sensitivity**: Participants were asked “Over the last week, sounds were too loud or uncomfortable for me when they seemed normal to others around me” and were asked to circle the number that represented where they fell on a range of 0 to 10 (0 = *No, not a problem*; 10 = *Yes, a very big problem*). Note this question was derived from the THS (Henry et al., 2015).

## Derived Measures and Variables

### Hearing Thresholds

- **BE PTA Low**: The average of the better ear thresholds at 500 Hz, 1 kHz, and 2 kHz (in dB HL), as measured from the Defense Occupational and Environmental Health Readiness System-Hearing Conservation (DOEHRS-HC, U.S. Army Public Health Center) hearing test using a CCA-200 audiometer (Benson Medical Instruments, Minneapolis, MN).
- **WE PTA Low**: The average of the worse ear thresholds at 500 Hz, 1 kHz, and 2 kHz (in dB HL).
- **BE PTA High**: The average of the better ear thresholds at 3 kHz, 4 kHz, and 6 kHz (in dB HL).
- **WE PTA High**: The average of the worse ear thresholds at 3 kHz, 4 kHz, and 6 kHz (in dB HL).
- **TTS Freq × Duration**: This was a derived variable obtained by multiplying the TTS Frequency score by the TTS Max Duration score. Both variables were on a 0 to 6 range, so the TTS Freq × Duration variable ranged from 0 to 36.

## Variable Distributions

### Demographic Data

Table 1 shows the mean and standard deviation values of the demographic answers to questions asked in the experiment. The cohort was, on average, about 34 years old and mostly male. Roughly 50% had been deployed to Iraq (Operation Iraqi Freedom; OIF) or Afghanistan (Operation Enduring Freedom; OEF), and about a quarter had reported being close to an explosive blast in training. Their hearing thresholds were, on average, relatively good, with a mean high-frequency pure-tone average (PTA) of 7.7 dB HL in the worse ear.

**Table 1.** Demographic and Environmental Variables in the Subject Population.

Variable	Value
Age	34.1 years (9.9)
Gender (Male)	81.7%
OIF/OEF Deployment	49.7%
Blast Far	43.6%
Blast Close	23.8%
mTBI Diagnosis	8.3%
> mTBI Diagnosis	1.4%
WE PTA Low	4.0 dB (4.5)
WE PTA High	7.7 dB (5.1)
BE PTA Low	0.2 dB (4.5)
BE PTA High	2.8 dB (5.8)

*Note*. The values are expressed in mean for each variable, with the standard deviations of each variable in parentheses. PTA = pure-tone average; mTBI = mild traumatic brain injury; WE PTA = worse ear pure-tone average; BE PTA = better ear pure-tone average; OIF/OEF Deployment = Operation Iraqi Freedom/Operation Enduring Freedom Deployment.

### Multiple Choice Responses

**Figure 1.**
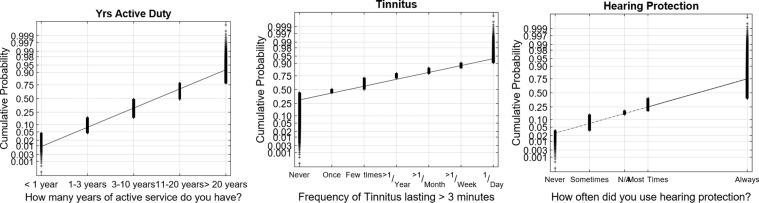
Normal probability plots of the distributions of responses for the number of years of active duty, the frequency of tinnitus, and the use of hearing protection. These plots show the cumulative distributions of the responses on a normal probability scale, so data points from a normal distribution will fall approximately on a straight line. The cumulative probability of the highest data point at each location on the *x* axis is the probability that an individual subject response was less than or equal to that value.

**Figure 2.**
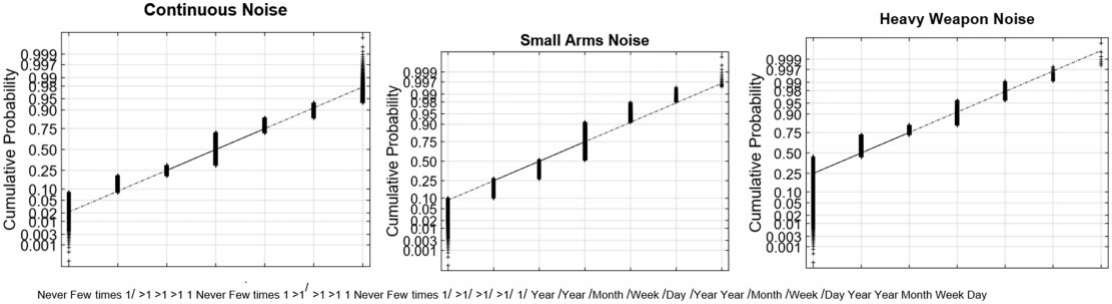
Normal plots of the distributions of responses for the questions relating to frequency of exposure to continuous, small-arms, and heavy weapon fire.

**Figure 3.**
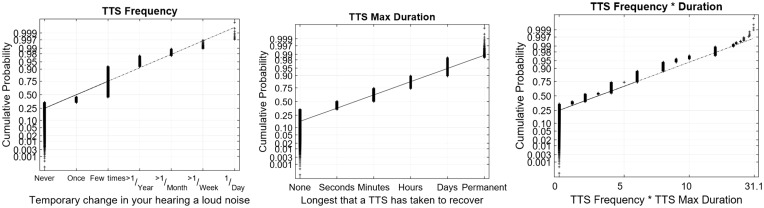
Normal plots of the distributions of responses for the frequency of temporary changes in hearing, the maximum duration for hearing to recover, and the derived TTS frequency × duration variable. TTS = temporary threshold shift.

**Figure 4.**
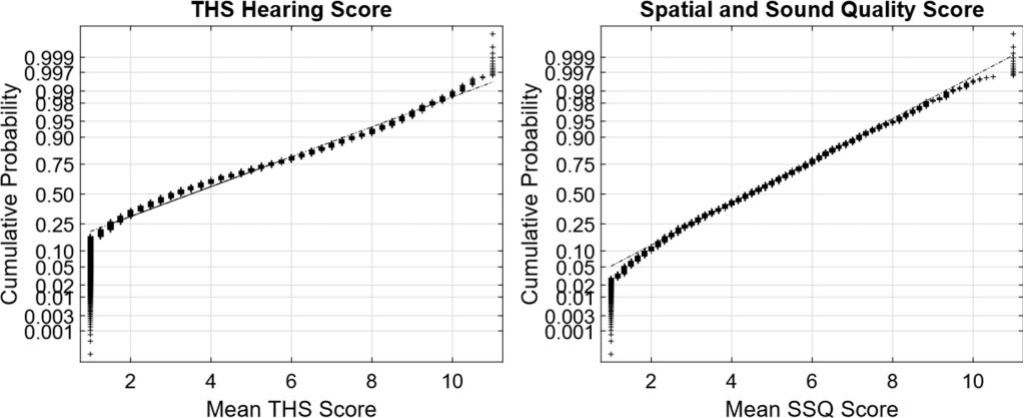
Normal plots of the distributions of responses for the THS and SSQ subjective hearing questionnaires. SSQ = Spatial and Sound Qualities; THS = tinnitus and hearing survey.

Figures 1 to 4 show normal quantile–quantile plots of the responses made for each of the multiple choice questions used in the experiment. In these plots, the ordinate shows the percentage of responses that were less than or equal to the value shown by the abscissa. The *y* axis is shown on a normal probability scale, which means that variables that fall on a straight line in the plot are exhibiting an approximately normal distribution.

Individual responses are shown by “+” symbols. Thus, the difference between cumulative probability for the highest “+” value in each category and the cumulative probability for the lowest “+” value in each category is an indicator of how many participants made each possible response. These distributions provide some useful insights into the noise exposure experience of the military population tested in this experiment. For example, in terms of perceptual tinnitus (second panel of Figure 1), roughly 44% of respondents reported they never experienced tinnitus lasting 3 minutes or longer, whereas roughly 29% of respondents said they experienced it once per year or more and 7% said they experienced it every day.

The results from the Hearing Protection questions (third panel of Figure 1) indicate that the SMs did generally wear hearing protection. These results show that only 3.5% of SMs reported that they never used hearing protection when they were exposed to noise. In comparison, 19% said that they used hearing protection “most times” and 60% said that they “always” used hearing protection.

The most frequent reported noise exposures occurred for Continuous Noise (first panel of Figure 2), with 70% of respondents reporting continuous noise exposure more than once per year and only 8% reporting they were never exposed to continuous noise.

In contrast, 48% of respondents reported Small Arms Fire more than once per year (second panel), and 10% reported no exposure to small arms fire. As expected, exposure to heavy weapon fire (third panel) was much less frequent, with only 18% reporting exposure at least once per year and 45% reporting no exposure.

Self-reported TTS was also relatively infrequent as shown in Figure 3. Moreover, 35% of respondents reported that they had never experienced a change in their hearing after a loud noise exposure, and only 8% said they had experienced them at least once per year. A plurality of respondents (47%) said they had experienced them a few times in their lifetime. Of those who did report a change in hearing, 24% of respondents said they took minutes to recover. Roughly 10% of respondents said that they had temporary hearing changes that took days to recover.

In almost all cases, the multiple-choice questions selected in this experiment produced response distributions that fell on or near a straight line in the normal distribution plot, which suggests that the responses can be viewed as discretely sampled values of variables with underlying Gaussian distributions. There are two notable exceptions. The first was the TTS Frequency × Duration value (Figure 3, third panel), which is the product of two Gaussians and therefore had a chi-squared distribution that gave it a long tail for the highest 1% to 2% of responses. This variable was normalized by replacing all values *x* greater than 12 with the value *log*(*x* − 11) + 12.

The second variable that was not intrinsically approximated by a normal distribution was the Hearing Protection question (Figure 1, third panel). The responses to this question (1–4) were transformed by replacing each value *x* with 1.7^*x*^ in order to produce the approximately normal distribution shown in the figure. This explains why the *x* axis spacing is nonlinear in that panel (recall that the N/A response for individuals who never wore any hearing protection was assigned to the mean value).

### Pure-Tone Averages

**Figure 5.**
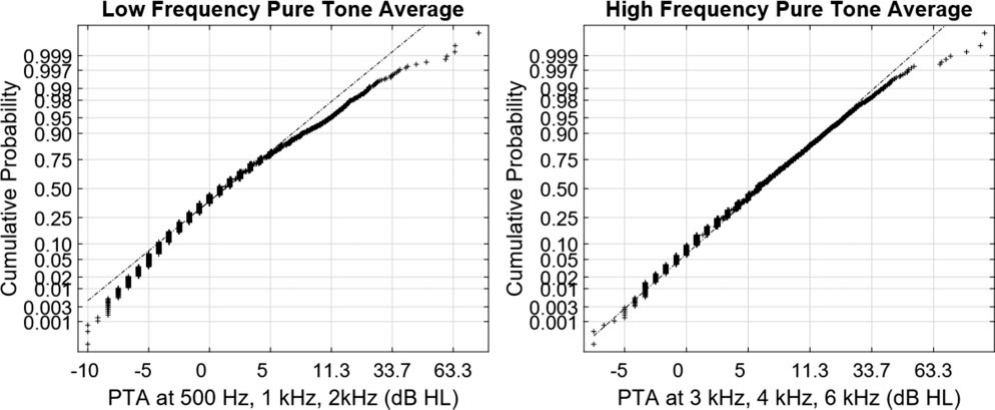
Normal plots of the distributions of the low- and high-frequency PTAs of all the subjects in the experiment. PTA = pure-tone average.

The PTAs of the participants were obtained from their annual hearing screenings and used to calculate Low-Frequency and High-Frequency PTAs for the left and right ears. In raw form, these PTAs were not normally distributed, so a transformation was applied to normalize them prior to their use in the regression analysis. This transformation replaced each PTA value greater than 9 dB HL *x* with *x*^0.65^ + (9 − 9^0.65^). The resulting distributions are shown in Figure 5.

## Data Analysis

The data from the study were analyzed using the statistics toolkit in MATLAB. Each analysis consisted of a set of predictor values and a single output variable selected from the data variables described in the previous section. First, a simple linear correlation was conducted between each predictor variable and the output variable. Next, a stepwise multivariate regression was performed. In this analysis, the predictor variables were added to the linear regression model of the output one variable at a time until no variables remained where the correlation with the residual variable had a *p* value less than .05. Finally, the relative contribution of each predictor value in the final model was determined by repeating the stepwise regression for all the variables in the model *excluding* that variable and determining how much the variance of the residual changed σout^2^ when that variable was excluded. This difference was expressed as a ratio σin^2^ in percent, with larger ratios indicating that the unique contribution that particular variable made to the overall model was greater than the unique contributions of the other variables.

## Results

### Predictors of TTS Frequency and Duration

The first question of interest was to identify the demographic and environmental exposure variable that best predicted the frequency and duration of self-reported TTS.

Table 2 shows the results of the regression analysis for predictors of the variable TTS Frequency. The “*r*” values in the second column of the table show the univariate correlations between each predictor value and the TTS Frequency. This analysis shows that all the predictor variables were significantly correlated with TTS Frequency.

**Table 2.** Stepwise Regression on the Variable TTS Frequency.

Variable	*r*	Order	Coeff	*p* Value	*σ_out_*^2^*/σ_in_*^2^ (%)
Continuous Noise	.302∗∗∗	1	0.163	.000	5.14
Blast Close	.270∗∗∗	2	0.343	.000	1.42
Heavy Weapon Noise	.263∗∗∗	4	0.077	.000	0.55
Years of Active Duty	.153∗∗∗	3	0.088	.000	0.52
Small Arms Noise	.245∗∗∗	5	0.073	.000	0.50
OIF/OEF Deployment	.175∗∗∗	6	0.116	.015	0.18
Hearing Protection	.049∗∗	–	–	.052	–
Blast Far	.263∗∗∗	–	–	.170	–
Gender	.101∗∗∗	–	–	.405	–
Age	.113∗∗∗	–	–	.619	–
**Total**	**.408**			*n* = 3,330	

*Note*. The second column shows the univariate correlation coefficient between each individual predictor variable and TTS frequency, with significance level shown by asterisks (* = *p* < .05; ** = *p* < .01; *** = *p* < .001). The “Order” column shows the order in which the variable was added to the multivariate regression, and the “*p Value*” column shows significance in the multivariate model. The last column shows the percent increase in the residual error when each variable was removed from the model. See text for details.

The right half of the table shows the results of the stepwise regression analysis. The “Order” column shows the order in which the variables were added to the regression. The “Coeff” column shows the coefficient of the variable, which by itself is not meaningful because the different predictor values represent different units. The *p* value column shows the *p* value of the component in the final stepwise regression model. Only those variables with *p* values less than .05 were retained in the final model, and these are shown in bold type. The last column shows the ratio of variance in the residual of the prediction when that variable was excluded or included in the model. This can be viewed as an estimate of the relative contribution each variable made to the prediction. Note that the predictor variables that were included in the final model are ranked in order of this ratio, and that variables that were not included in the model are ranked in order of increasing *p* value.

The bottom row of the table shows the correlation coefficient (*r*) of the final prediction, which in this case was .408.

The results of this analysis show that Continuous Noise was the strongest predictor of TTS Frequency, with a variance ratio almost three times as large as the second strongest predictor, which was Blast Close. Heavy Weapon Noise Exposure, Small Arms Noise Exposure, and Years of Active Duty service all made similar contributions, with Combat Deployment making a very small additional contribution.

Table 3 shows a similar analysis for the variable TTS Duration. Again, Continuous Noise was the strongest predictor. However, in the prediction of the Maximum TTS duration, Age was the second strongest predictor. Blast Close, Small Arms Noise, and Blast Far all made lesser contributions to the overall prediction.

**Table 3.** Stepwise Regression on the Variable of TTS Duration.

Variable	*r*	Order	Coeff	*p* Value	*σ_out_*2*/σ_in_*2 (%)
Continuous Noise	.241∗∗∗	2	0.150	.000	2.73
Age	.157∗∗∗	3	0.019	.000	1.86
Blast Close	.291∗∗∗	1	0.476	.000	1.42
Small Arms Noise	.239∗∗∗	4	0.133	.000	1.13
Blast Far	.279∗∗∗	5	0.254	.000	0.52
OIF/OEF Deployment	.190∗∗∗	–	–	.197	–
Years of Active Duty	.171∗∗∗	–	–	.272	–
Heavy Weapon Noise	.240∗∗∗	–	–	.313	–
Hearing Protection	.071∗∗∗	–	–	.734	–
Gender	.086∗∗∗	–	–	.924	–
**Total**	**.392**			*n* = 3,330	

*Note*. See Table 2 for details.

Table 4 shows the results for prediction of the multiplicative variable TTS Frequency × TTS Duration. In this combined variable, it is clear that frequent exposure to Continuous Noise was the strongest predictor, followed by Blast Close, Small Arms Noise, and Heavy Weapon Noise. Age and Years of Service made small additional contributions to the predictions.

**Table 4.** Stepwise Regression on the Variable of TTS Freq × Duration.

Variable	*r*	Order	Coeff	*p* Value	*σ_out_*^2^ */σ_in_*^2^ (%)
Continuous Noise	.288∗∗∗	2	0.425	.000	4.18
Blast Close	.289∗∗∗	1	1.187	.000	2.10
Small Arms Noise	.265∗∗∗	3	0.316	.000	1.12
Heavy Weapon Noise	.263∗∗∗	5	0.186	.000	0.39
Age	.135∗∗∗	4	0.024	.014	0.18
Years of Active Duty	.163∗∗∗	6	0.189	.026	0.15
Hearing Protection	.054∗∗	–	–	.064	–
Blast Far	.269∗∗∗	–	–	.186	–
OIF/OEF Deployment	.172∗∗∗	–	–	.442	–
Gender	.095∗∗∗	–	–	.857	–
**Total**	.**415**			*n* = 3,330	

*Note*. See Table 2 for details.

Overall, these results suggest that frequent exposure to loud continuous noise is the greatest risk factor associated with experiencing frequent or severe TTS, and exposure to a nearby blast is a distant second. Exposure to Small Arms or Heavy Weapons Noise were also significant predictors of TTS, but they were not as strongly associated with TTS as Continuous Noise and Nearby Blast. Age and Years of Duty also made a contribution, possibly indicating there is a certain risk of experiencing a severe TTS every year and that the cumulative risk of experiencing such an acoustic trauma increases with time.

### Predictions of Hearing Thresholds

Because PTS is the gold standard for evaluating hearing injury, it is also helpful to examine the extent to which the different environmental and demographic factors in the survey predicted the pure-tone thresholds of the participants. Table 5 shows the predictive values for the Low-Frequency (500 Hz, 1 kHz, 2 kHz) PTAs of the worse ear.

**Table 5.** Stepwise Regression on Low-Frequency PTA Threshold in the Worse Ear.

Variable	*r*	Order	Coeff	*p* Value	*σ_out_*^2^*/σ_in_*^2^ (%)
Age	.179∗∗∗	2	0.092	.000	2.73
TTS Freq × Dur	.207∗∗∗	1	0.447	.000	1.74
Continuous Noise	.103∗∗∗	4	0.208	.000	0.56
TTS Max Duration	.165∗∗∗	3	−0.509	.000	0.43
Hearing Protection	−.041∗	5	−0.108	.001	0.31
Gender	.070∗∗∗	7	0.556	.005	0.24
OIF/OEF Deployment	.085∗∗∗	6	−0.449	.025	0.15
Blast Far	.034	8	−0.372	.028	0.15
TTS Frequency	.199∗∗∗	–	–	.079	–
Years of Active Duty	.161∗∗∗	–	–	.409	–
Heavy Weapon Noise	.022	–	–	.449	–
Small Arms Noise	.025	–	–	.565	–
Blast Close	.061∗∗∗	–	–	.879	–
**Total**	.**289**			*n* = 3,330	

*Note*. See Table 2 for details. TTS = temporary threshold shift.

These predictions (and those for better ear, which are not shown), indicate that Age and TTS Freq × Dur, respectively, were the strongest predictors of the Low-Frequency PTAs, with variance ratios 2.5 to 5 times as high as those measured for Continuous Noise. The use of Hearing Protection was also a significant factor, indicating that those who reported that they frequently used hearing protection were less likely to have elevated low-frequency thresholds than those who did not.

There was also a contribution of TTS Max Duration. Note, however, that the coefficient for this variable was opposite in sign from the TTS Freq × Dur Variable, indicating that it was correcting for an overestimation of the influence of duration in the combined TTS Freq × Dur. If a single variable of TTS Freq × Dur − TTS Dur was included in the regression, it was the only significant TTS variable and its variance ratio increased roughly 50%. This suggests that TTS is the most dominant predictor of low-frequency hearing loss in this population of SMs.

Gender made a small contribution to both ears, with slightly higher thresholds for males than for females.

OIF/OEF deployment also made a slight contribution to both ears, but, somewhat counterintuitively, the sign of the coefficient was negative, indicating that individuals deployed in OIF/OEF had *better* low-frequency thresholds than those who were not. The reasons for this are unclear.

Table 6 shows the predictive values for the High-Frequency (3, 4, and 6 kHz) PTAs of the worse ear. In these predictions, and in those for the High-Frequency PTA of the better ear (which are not shown), the most relevant factors were Age, Gender, TTS Freq × Dur, Continuous Noise, and Hearing Protection, in that order. In these predictions of high-frequency hearing loss, Age was by far the most dominant factor, with variance ratios 7 to 11 times higher than Gender, which was the second strongest predictor. The variables related to noise exposure explained relatively less of the variance but, again, the contribution of TTS Freq × Dur (as measured by variance ratio) was roughly 4 times larger than that of Continuous Noise.

**Table 6.** Stepwise Regression for the High-Frequency PTA Threshold in the Worse Ear.

Variable	*r*	Order	Coeff	*p* Value	*σ_out_*^2^ */σ_in_*^2^ (%)
Age	.439∗∗∗	1	0.212	.000	22.21
Gender	.195∗∗∗	2	2.057	.000	3.30
TTS Freq × Dur	.228∗∗∗	3	0.230	.000	2.59
Continuous Noise	.070∗∗∗	4	0.128	.014	0.18
Hearing Protection	−.002	5	−0.077	.024	0.15
Years of Active Duty	.393∗∗∗	–	–	.134	–
OIF/OEF Deployment	.309∗∗∗	–	–	.143	–
Blast Close	.174∗∗∗	–	–	.312	–
Small Arms Noise	.059∗∗∗	–	–	.338	–
TTS Max Duration	.216∗∗∗	–	–	.347	–
Heavy Weapon Noise	.063∗∗∗	–	–	.663	–
TTS Frequency	.198∗∗∗	–	–	.853	–
Blast Far	.136∗∗∗	–	–	.903	–
**Total**	.**499**			*n* = 3,330	

*Note*. See Table 2 for details. TTS = temporary threshold shift.

### Predictions of Perceptual Tinnitus

The analysis of tinnitus is generally separated into perceptual tinnitus, where the listener perceives a phantom tone, buzz, or other sound, and bothersome tinnitus, when the phantom sound becomes distracting enough to interfere with daily activities. In this study, we only looked at perceptual tinnitus, which was defined by how frequently the participant reported hearing buzzing or ringing in the ear that lasted for more than 3 minutes.

Table 7 shows the results of the stepwise regression on this perceptual tinnitus variable. Here, the responses were clearly dominated by TTS Freq × Dur, which had a variance ratio almost ten times as large as Years of Active Duty, which was the second strongest predictor. Other factors that were less predictive, but still significant, included the better ear PTA thresholds, Continuous Noise, Close Blast Exposure, and Small Arms Noise.

**Table 7.** Stepwise Regression for the Frequency of Perceptual Tinnitus.

Variable	*r*	Order	Coeff	*p* Value	*σ_out_*^2^ */σ_in_*^2^ (%)
TTS Freq × Dur	.476∗∗∗	1	0.228	.000	15.39
Years of Active Duty	.247∗∗∗	3	0.214	.000	1.55
BE PTA High	.300∗∗∗	2	0.040	.000	1.15
Continuous Noise	.230∗∗∗	4	0.105	.000	0.75
BE PTA Low	.247∗∗∗	5	0.035	.000	0.62
Blast Close	.237∗∗∗	6	0.246	.001	0.31
Small Arms Noise	.195∗∗∗	7	0.075	.007	0.22
WE PTA High	.297∗∗∗	–	–	.070	–
mTBI Diagnosis	.139∗∗∗	–	–	.081	–
Heavy Weapon Noise	.161∗∗∗	–	–	.099	–
Age	.226∗∗∗	–	–	.126	–
> mTBI Diagnosis	.060∗∗∗	–	–	.144	–
Blast Far	.194∗∗∗	–	–	.229	–
WE PTA Low	.241∗∗∗	–	–	.249	–
OIF/OEF Deployment	.193∗∗∗	–	–	.391	–
TTS Max Duration	.441∗∗∗	–	–	.630	–
TTS Frequency	.421∗∗∗	–	–	.645	–
Gender	.096∗∗∗	–	–	.809	–
Hearing Protection	.048∗∗	–	–	1.000	–
**Total**	.**545**			*n* = 3,330	

*Note*. See Table 2 for details. PTA = pure-tone average; mTBI = mild traumatic brain injury; WE PTA = worse ear pure-tone average; BE PTA = better ear pure-tone average; TTS = temporary threshold shift; OIF/OEF Deployment = Operation Iraqi Freedom/Operation Enduring Freedom Deployment

### Predictions of Hearing Difficulties

We contend that the goal of a hearing conservation program is to avoid hearing damage that results in a degradation in overall quality of life, regardless of whether that degradation is accompanied by an associated increase in pure-tone threshold. Under this model, some of the most meaningful outcome variables are the subjective hearing complaints experienced by SMs who are noise exposed. We used four different instruments to assess hearing complaints in this study. Table 8 shows the stepwise prediction of the mean score on the hearing-related questions of the Tinnitus and Hearing Survey. In this analysis, the variable TTS Freq × Dur was by far the most dominant predictor, with a variance ratio five times as large as the second strongest predictor, the Better-Ear High-Frequency PTA. Continuous Noise contributed roughly the same as the Better Ear High-Frequency PTA, with Years of Active Duty, the Worse Ear PTA, and Gender making smaller contributions to the overall prediction.

**Table 8.** Stepwise Regression for the Composite Hearing Score on the Tinnitus and Hearing Survey.

Variable	*r*	Order	Coeff	*p* Value	*σ_out_*^2^ */σ_in_*^2^ (%)
TTS Freq × Dur	.434∗∗∗	1	0.252	.000	12.91
BE PTA High	.332∗∗∗	2	0.074	.000	2.55
Continuous Noise	.245∗∗∗	3	0.215	.000	2.26
Years of Active Duty	.238∗∗∗	4	0.241	.000	1.30
WE PTA Low	.267∗∗∗	5	0.047	.000	0.71
Gender	.056∗∗	6	−0.247	.012	0.19
Small Arms Noise	.165∗∗∗	–	–	.055	–
Blast Far	.182∗∗∗	–	–	.066	–
Hearing Protection	.008	–	–	.098	–
mTBI Diagnosis	.122∗∗∗	–	–	.127	–
Blast Close	.192∗∗∗	–	–	.132	–
TTS Frequency	.391∗∗∗	–	–	.269	–
TTS Max Duration	.390∗∗∗	–	–	.289	–
BE PTA Low	.266∗∗∗	–	–	.306	–
WE PTA High	.313∗∗∗	–	–	.564	–
Age	.211∗∗∗	–	–	.630	–
Heavy Weapon Noise	.144∗∗∗	–	–	.636	–
> mTBI Diagnosis	.036∗	–	–	.655	–
OIF/OEF Deployment	.178∗∗∗	–	–	.718	–
**Total**	.**527**			*n* = 3,330	

*Note*. See Table 2 for details. PTA = pure-tone average; mTBI = mild traumatic brain injury; WE PTA = worse ear pure-tone average; BE PTA = better ear pure-tone average; TTS = temporary threshold shift; OIF/OEF Deployment = Operation Iraqi Freedom/Operation Enduring Freedom Deployment.

Table 9 shows the stepwise predictions for the SSQ questionnaire. Here, the predictions were generally not quite as strong, and, although TTS × Freq was still the strongest predictor, its role was not as dominant as on the THS survey. As in the THS, the two hearing thresholds that contributed were the High-Frequency PTAs in the *better* ear and the Low-Frequency PTAs in the *worse* ear. However, with the SSQ, there was a significant contribution of TBI diagnosis, with those diagnosed with more than a mild TBI scoring, on average, almost one full point (0.908) worse on the SSQ. There was also a significant effect of hearing protection, with improved SSQ scores for those who reported using hearing protection more often.

**Table 9.** Stepwise Regression for the Composite SSQ Score.

Variable	*r*	Order	Coeff	*p* Value	*σ_out_*^2^*/σ_in_*^2^ (%)
TTS Freq × Dur	.292∗∗∗	1	0.179	.000	1.47
Years of Active Duty	.174∗∗∗	4	0.329	.000	1.21
BE PTA High	.240∗∗∗	2	0.042	.000	1.14
WE PTA Low	.224∗∗∗	3	0.045	.000	0.84
Age	.123∗∗∗	5	−0.026	.000	0.52
> mTBI Diagnosis	.073∗∗∗	6	0.908	.001	0.34
Continuous Noise	.141∗∗∗	7	0.069	.002	0.29
Hearing Protection	−.029	8	−0.044	.002	0.28
TTS Max Duration	.251∗∗∗	9	−0.115	.048	0.12
Gender	.038∗	–	–	.103	–
mTBI Diagnosis	.111∗∗∗	–	–	.112	–
Small Arms Noise	.103∗∗∗	–	–	.163	–
Blast Far	.114∗∗∗	–	–	.296	–
OIF/OEF Deployment	.128∗∗∗	–	–	.472	–
WE PTA High	.222∗∗∗	–	–	.613	–
BE PTA Low	.215∗∗∗	–	–	.658	–
TTS Frequency	.265∗∗∗	–	–	.663	–
Heavy Weapon Noise	.093∗∗∗	–	–	.791	–
Blast Close	.113∗∗∗	–	–	.957	–
**Total**	.**381**			*n* = 3,330	

*Note*. See Table 2 for details. PTA = pure-tone average; mTBI = mild traumatic brain injury; WE PTA = worse ear pure-tone average; BE PTA = better ear pure-tone average; TTS = temporary threshold shift; OIF/OEF Deployment = Operation Iraqi Freedom/Operation Enduring Freedom Deployment.

Tables 10 and 11 show predictions for the questions related to sound localization and sound sensitivity. The predictors for sound sensitivity were very similar to those for the SSQ. For sound localization, the predictors were also similar to the SSQ except that the factors related to Age and TBI did not play a role.

**Table 10.** Stepwise Regression for Response on Question Evaluating Subjective Localization Ability.

Variable	*r*	Order	Coeff	*p* Value	*σ_out_*^2^*/σ_in_*^2^ (%)
TTS Freq × Dur	.217∗∗∗	1	0.159	.000	1.29
BE PTA High	.197∗∗∗	3	0.040	.000	1.17
Gender	–.049**	4	−0.447	.000	0.89
WE PTA Low	.199***	2	0.042	.000	0.80
Hearing Protection	−.068∗∗∗	5	−0.053	.000	0.44
TTS Max Duration	.180∗∗∗	6	−0.135	.016	0.17
WE PTA High	.197∗∗∗	–	–	.051	–
Blast Far	.073∗∗∗	–	–	.068	–
Continuous Noise	.084∗∗∗	–	–	.068	–
Small Arms Noise	.048∗∗	–	–	.073	–
Age	.065∗∗∗	–	–	.095	–
Years of Active Duty	.104∗∗∗	–	–	.135	–
TTS Frequency	.202∗∗∗	–	–	.141	–
Heavy Weapon Noise	.064∗∗∗	–	–	.153	–
> mTBI Diagnosis	.033	–	–	.191	–
Blast Close	.076∗∗∗	–	–	.200	–
OIF/OEF Deployment	.073∗∗∗	–	–	.355	–
mTBI Diagnosis	.056∗∗	–	–	.549	–
BE PTA Low	.184∗∗∗	–	–	.877	–
**Total**	.**307**			*n* = 3,330	

*Note*. See Table 2 for details. PTA = pure-tone average; mTBI = mild traumatic brain injury; WE PTA = worse ear pure-tone average; BE = better ear pure-tone average; TTS = temporary threshold shift; OIF/OEF Deployment = Operation Iraqi Freedom/Operation Enduring Freedom Deployment.

**Table 11.** Stepwise Regression for Response on Question Evaluating Subjective Sensitivity to Loud Sounds.

Variable	*r*	Order	Coeff	*p* Value	*σ_out_*^2^*/σ_in_*^2^ (%)
TTS Freq × Dur	.223∗∗∗	1	0.126	.000	2.37
Age	.108∗∗∗	4	0.023	.000	0.79
Continuous Noise	.144∗∗∗	3	0.152	.000	0.76
Hearing Protection	−.047∗∗	5	−0.089	.000	0.62
WE PTA Low	.129∗∗∗	2	0.039	.000	0.49
Gender	−.019	6	−0.424	.000	0.43
Small Arms Noise	.097∗∗∗	7	0.126	.002	0.29
> mTBI Diagnosis	.057∗∗	8	0.887	.012	0.19
TTS Max Duration	.189∗∗∗	–	–	.064	–
Blast Far	.112∗∗∗	–	–	.087	–
Years of Active Duty	.117∗∗∗	–	–	.094	–
TTS Frequency	.210∗∗∗	–	–	.131	–
OIF/OEF Deployment	.100∗∗∗	–	–	.206	–
mTBI Diagnosis	.081∗∗∗	–	–	.388	–
WE PTA High	.104∗∗∗	–	–	.635	–
Blast Close	.086∗∗∗	–	–	.791	–
BE PTA Low	.118∗∗∗	–	–	.906	–
Heavy Weapon Noise	.087∗∗∗	–	–	.931	–
BE PTA High	.112∗∗∗	–	–	.968	–
**Total**	.**284**			*n* = 3,330	

*Note*. See Table 2 for details. PTA = pure-tone average; mTBI = mild traumatic brain injury; WE PTA = worse ear pure-tone average; BE PTA = better ear pure-tone average; TTS = temporary threshold shift; OIF/OEF Deployment = Operation Iraqi Freedom/Operation Enduring Freedom Deployment.

### Relationship Between Audiogram Shape and TTS

Although the data clearly show that there is a strong relationship between self-reported incidents of TTS and subjective hearing complaints, there is certainly some room for concern that this relationship might be explained by psychological factors related to the way the questions were asked. For example, one possibility is that individuals with hearing complaints might be more likely to remember temporary hearing changes from the past than those who do not have any such complaints. Or, alternatively, people who are more sensitive to or annoyed by hearing problems might be more likely to notice temporary hearing changes when they occur.

Although these psychological factors almost certainly played some role in the data from this experiment, there is also some objective evidence from the shape of the audiograms that suggests that there were systematic physiological differences in the hearing profiles of individuals who reported TTS and those that did not.

**Figure 6.**
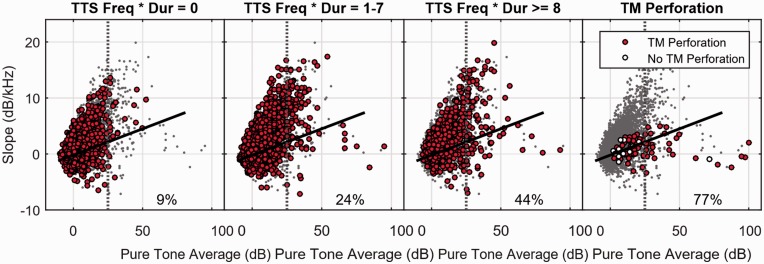
Plots showing the relationship between PTA and Slope of the 6,660 individual audiograms tested in the study. The black lines are a fit to the 25th percentile slope values of the normal audiograms, and the numbers in the bottom right of each panel show the percentage of audiograms for hearing impaired listeners (PTA > 25 dB) that fell below this 25th percentile line. The rightmost panel compares the data of the present study to data from Littlefield & Brungart (2019). See text for details. TTS = temporary threshold shift; TM = tympanic membrane.

The individual data points plotted in Figure 6 show the relationship between slope and PTA for each of the 6,660 ears evaluated as part of the experiment. For each ear, the slope (in dB/kHz) was calculated by fitting a line to the pure-tone audiogram for the six frequencies from 500 Hz to 6 kHz. Then the PTA was calculated simply by taking the average threshold across the six frequencies. In the first panel, the filled circles show the data points where the SM never reported experiencing any kind of temporary hearing change (i.e., TTSFreq × Dur = 0). In the second panel, the filled circles show data points where the SMs reported a moderate level of TTS (TTSFreq × Dur = 1–7). In the third panel, the filled circles show data points where SMs reported a more substantial level of TTS (TTSFreq × Dur ≥ 8). Finally, in the fourth panel, we show data from Littlefield and Brungart (2019) for the audiograms of 98 ears from SMs who were close enough to an explosive blast to puncture at least one tympanic membrane (TM). The closed circles show 83 ears with a punctured TM, and the open circles show 15 ears that were contralateral to an ear with a punctured TM but were not punctured. For reference, all of the other data points are repeated in each panel as gray dots.

The dark black lines in the figure divide the audiograms in the normal hearing range into those that fall above the 25th percentile in slope for a given PTA value (above the line) and those than fall below the 25th percentile in slope for a given PTA value (below the line). These lines, which are the same in each panel, were calculated by dividing the normal hearing audiograms into five 10-dB wide bins centered at PTA values of 0, 5, 10, 15, and 20 dB HL. The 25th percentile value for each bin was calculated, and a line was fit to these 25th percentile values and extended over the region from −10 dB and up to +80 dB.

In the normal hearing range (PTA < 25 dB), all three TTS regions had roughly the same percentage of data points (approximately 25%) under the 25th percentile line. However, in cases where there was some hearing loss (i.e., to the right of the vertical dotted line at 25 dB), there were substantial differences in the pattern of slopes. In the case where there was no reported TTS (first panel), only 9% of the data points for hearing impaired listeners were under the line. In the moderate TTS case (second panel), 24% were under the line. In the severe TTS case (third panel), 44% were under the line. In the data for individuals who had at least one ear's TM punctured by explosive blast (third panel), 77% of the data points were under the line. Thus, it appears that hearing impaired listeners who report a history of TTS tend to have substantially flatter audiograms (lower slopes at a given PTA) than those who do not report a history of TTS, and that this shift appears to be on a trajectory in the direction of ears that suffered extreme trauma from exposure to explosive blast.

A further analysis indicated that the change in audiogram shape seen for hearing impaired SMs with a history of TTS was more strongly related to the frequency of the TTS than its reported duration. Only 19% of hearing impaired SMs who reported experiencing TTS no more than a few times in their lifetime had audiograms that fell under the line in Figure 6. In contrast, 50% of hearing impaired SMs who reported one or more TTS per year had audiograms that fell in this region. When the results were analyzed to account for the maximum duration of the TTS rather than its frequency, there was no significant increase in the number of hearing impaired audiograms falling under the 25th percentile line until the SM reported a permanent change in hearing.

Although these data are not completely conclusive, they do provide some validation that the subjective reports of TTS history obtained in this survey were correlated with objective changes in auditory function that were consistent with what we have seen in military populations with confirmed blast exposures that resulted in perforated TMs (Littlefield & Brungart, 2019).

## Discussion

### The Importance of Subjective TTS in Predicting Subjective Hearing Handicap

The purpose of this study was to use a brief survey administered to SMs to assess the relationship between the different types of occupational noise exposures, hearing thresholds, and subjective hearing complaints. The results of the study show that self-reports of temporary hearing loss are substantially stronger predictors of perceptual tinnitus and subjective hearing difficulty than self-reported exposure to continuous, small arms, or heavy weapon noise.

**Figure 7.**
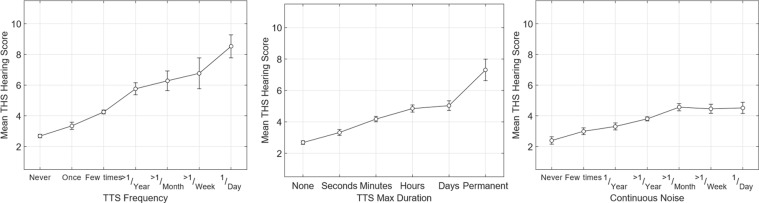
Plots of the mean score on the THS Hearing questions as a function of self-reported TTS Frequency (first panel), TTS Duration (second panel), and continuous noise exposure (third panel). Error bars shows the 95% confidence intervals around each data point. TTS = temporary threshold shift; THS = tinnitus and hearing survey.

Self-reported TTS was also as good as or better than self-reported noise exposure for predicting hearing thresholds. This is illustrated in Figure 7, which plots the average THS hearing score as a function of three variables: TTS Freq, TTS Dur, and Continuous Noise exposure. Of the three plots, the one for TTS Freq (first panel) shows the strongest relationship between exposure and handicap, with an increase in THS for even a single reported TTS and a large increase in the THS value when the TTS frequency increased to once per year or more.

The effect of the maximum reported TTS duration (second panel) was more modest, except for the case where the participants reported a single noise exposure that resulted in a permanent change in hearing. It is notable both that there was a substantial increase in THS score even for those reporting TTSs that resolved within minutes, and that there was not much difference in THS score for those with TTS durations that lasted for hours versus those that lasted for days.

The results for Continuous Noise exposure (third panel) are notable primarily in terms of their comparison to the TTS exposures. Individuals who reported experiencing TTS more than once per year had substantially worse THS values than those who reported daily exposure to continuous noise. Individuals who had at least one TTS lasting hours had worse THS scores than those exposed to noise every day. For those exposed to continuous noise, there was not much difference between those exposed monthly and those exposed weekly or daily.

Certainly, there are some limitations and caveats associated with these results. Because of our time limitations, our noise exposure questions were somewhat rudimentary, and it is possible that a more comprehensive or better constructed set of noise questions might have increased the apparent contribution of noise exposure to apparent handicap. Also, as was mentioned earlier, it is possible that the sensitivity of THS to reported subjective TTS could represent some underlying confounds. For example, it is possible that people who are more aware of or pay more attention to their hearing would be more likely to notice a TTS after loud noise exposure, and that these same people might also be more likely to report a hearing handicap on a survey like the THS.

The results also appear to contradict the results of Le Prell et al. (2018), who found no systematic difference between functional measures of hearing in individuals who indicated a past history of noise-induced hearing changes and those who did not report experiencing any past noise-related changes in hearing.

Nevertheless, it is hard to dismiss the completely dominant role that questions related to TTS appeared to play in predicting virtually all of the subjective measures of hearing complaint, or the fact that the TTS questions were stronger predictors of hearing thresholds than the other questions related to noise exposure. It is also difficult to believe that the systematic relationship between audiogram shape and subjective TTS does not reflect some underlying link between self-perceived TTS and hearing damage.

Thus, on the basis of these results, we would strongly recommend adding questions regarding TTS to future studies of noise exposure. Note that this recommendation is based on the important role that subjective TTS might have on predicting the *consequences* of noise exposure, rather than the assumption that TTS is somehow a more reliable way of assessing quantitative noise dose than a task-based exposure study like the NEQ or NESI. Although we are aware of very few noise surveys that have included questions on TTS, there were three questions very similar to our TTS questions included as potential screening questions in the development of the NEQ. These questions asked about the frequency of exposure to sounds that made your hearing “ring or buzz,” “sound muffled,” or “feel full or hurt.” Ultimately, these three questions were rejected from use in the final 1-minute NES screener because they were not correlated with the Annual Noise Exposure predicted by the full NEQ (Johnson et al., 2017).

In our study, there was some positive correlation between the self-reported noise exposures and the frequency and duration of TTS. It may be the case that this greater correlation is a result of the higher noise exposures one might expect to see in a military population versus the population of 114 college freshman and 59 civilian adults used in the validation of the NEQ (Johnson et al., 2017). However, it may be even more likely that the prevalence of TTS was an indicator of individuals who were experiencing a stronger biological response to a given noise dose, which ultimately made them more likely to experience tinnitus and to suffer from subjective hearing impairments than those who received the same noise dose but did not experience TTS.

It is also worth noting some potential differences between this study and the study by Le Prell et al. (2018) that also looked at self-report of temporary noise-induced hearing loss and found no correlations with long-term hearing function. One key difference may have been the wording of the question. Our survey specifically asked about a *change in hearing* and a “dullness” or “muffled” quality, whereas theirs specifically asked about a *hearing loss*, which might be interpreted more as an inability to hear quiet sounds and might have been harder to judge subjectively. The earlier study also did not look at subjective measures of hearing difficulty, which are probably only meaningful in studies that incorporating large cohorts of participants with and without self-reported noise-related changes in hearing. These differences might help explain why LePrell et al. did not find self-reported hearing changes to be a useful predictor of hearing problems in their population.

### The Relative Roles of Continuous Noise, Impulsive Noise, and Blast Exposure on Hearing Problems in the Military

In general, the results of this study show that questions that inquire about the frequency and duration of perceived temporary shifts in hearing after noise exposure are stronger predictors of hearing handicap than questions that more directly ask about the frequency and type of noise exposure experienced by the listener. However, it is important to note that these environmental noise factors are strong predictors of the level of TTS. They are also significant predictors of permanent shifts in the Low- and High-Frequency PTA, even when the TTS variables are accounted for. So the results should not be interpreted to mean that noise and blast exposure are not important to determining hearing handicap in the military. Rather, they suggest that perceived temporary shifts in hearing are good indicators that hearing damage may be occurring across a variety of noise sources.

### The Importance of Better and Worse Ear Thresholds in the High- and Low-Frequency Regions

One interesting finding in the results for subjective hearing complaint is that perceived hearing handicap was generally predicted by the *High-Frequency* PTA in the *better* ear, but by the *Low-Frequency* PTA in the *worse* ear. In most tasks, one would expect the hearing threshold in the better ear to be a stronger driver of overall hearing ability, as it is going to determine the audibility of the low-level components of the signal. However, in tasks that require binaural processing of low-frequency temporal fine structure, such as sound localization (Wightman & Kistler, 1992) or spatial release from masking (Levitt & Rabiner, 1967), performance might be determined by the ear with the worst threshold.

### Evidence for the Importance of Hearing Protection Use

Although it was a relatively minor predictor, it is worth noting that increased self-reported use of hearing protection was associated with significant improvements in the high- and low-frequency thresholds, the SSQ score, sound localization, and sound sensitivity. These findings provide further support for the importance of hearing protection use in military hearing conservation programs.

## Conclusions

This study focused on evaluating the relationship between subjective reports about noise exposure, audiometric thresholds, and subjective complaints about hearing problems experienced by SMs. The main finding of the study was that self-reported temporary changes in hearing was the strongest predictor of all the subjective hearing problems evaluated in the study, and that it was stronger than other noise-based questionnaires for predicting both high- and low-frequency thresholds.

On the basis of these findings, we think it would be prudent to reassess current military and civilian hearing conservation programs to increase the emphasis given to avoiding TTS events, rather than simply reducing overall noise dose. Individuals who are experiencing several noticeable TTSs per year appear to be at very high risk of developing tinnitus and hearing difficulties, even if those TTSs only last a few minutes, and even if pure-tone hearing thresholds continue to fall in the normal range. Thus, we do not think it would be unreasonable to include information related to this finding in the training provided in hearing conservation programs. In particular, we think SMs should be told that it is not normal or acceptable to experience these kinds of transient changes in hearing, and that those who do experience them should be encouraged to engage with their audiologist or hearing program officer to explore possible interventions, which might include the use of alternative hearing protection options or a change in occupational or recreational behaviors that are resulting in subjective hearing changes.

On a broader scale, these results appear to provide some support for the notion that noise exposures that result in temporary hearing shifts can increase the probability of tinnitus and hearing difficulties even when they do not result in significant permanent changes in hearing thresholds. Many recent studies have looked for a relationship between noise exposure and suprathreshold hearing deficits, and the results have generally been mixed (Grinn et al., 2017; Guest, Munro, Prendergast, Millman, & Plack, 2018b; Le Prell et al., 2018; Liberman, Epstein, Cleveland, Wang, & Maison, 2016). However, this study differs from those early studies in a number of ways. First, it includes a population that may generally be more noise exposed than the civilian population, particularly to impulsive and blast noise. Second, it includes individuals with some level of mild hearing loss (i.e., elevated hearing thresholds) and controls for these losses, rather than attempting to restrict recruitment only to individuals with clinically normal hearing. Finally, it asked specifically about perceived hearing changes, rather than simply attempting to estimate the amount of noise exposure received by the participants in the study. As mentioned earlier, this may better account for individual differences in *susceptibility* to hearing damage.

Note that many of the scientific foundations for identifying cochlear synaptopathy are based on animal models that have very little genetic diversity (Kujawa & Liberman, 2009). In these populations, a given noise dose will generate the same response in every animal, so there is no need to take account of individual variations in susceptibility when evaluating the effects of noise exposure on the cochlea. However, in the human population, there may be a very wide variability in susceptibility to noise exposure. Thus, it may not be surprising that studies that focus only on estimates of noise dose might fail to find systematic noise exposure effects. Although undeniably crude, the subjective TTS questions used in this study might indeed do a better job of estimating susceptibility to noise exposure than the much more sophisticated instruments targeted at accurately estimating noise dose that have been used in other studies of hidden hearing loss in the human population.

This study has provided some preliminary insights into the role of TTS on hearing deficits in the military, but many questions remain unanswered. Further research is now needed to determine (a) what the sources of the TTSs were, and whether that has an effect on outcome and (b) whether there are other objective measures, besides PTA and audiogram shape, that correlate with perceived TTS. This might include objective performance measures, such as speech in noise performance, or objective physiological measures, such as DPOAEs and auditory brainstem response (ABR) Wave I amplitudes. In fact, at least one study has already reported some evidence for slightly better DPOAEs and WAVE I ABRs in patients who reported no history of noise-induced changes in hearing, although this was not accompanied with any differences in perceived handicap or functional performance (Fulbright, 2016). A better understanding of factors that determine individual susceptibility and sensitivity to temporary hearing changes could provide valuable insights into the underlying mechanisms of noise-induced hearing damage and, in particular, those mechanisms that might lead to reduced functional performance without an accompanying increase in the pure-tone audiogram. However, even in the absence of additional knowledge about these underlying mechanisms, the results of this study suggest that placing a greater emphasis on minimizing events that result in temporary changes in subjective hearing might improve the effectiveness of hearing conservation programs in the military and, ultimately, improve the long-term hearing outcomes of our SMs and veterans.

## References

[bibr1-2331216519872601] Bramhall N. F. Konrad-Martin D. McMillan G. P. Griest S. E. (2017) Auditory brainstem response altered in humans with noise exposure despite normal outer hair cell function. Ear and Hearing 38(1): e1 . doi: 10.1097/AUD.0000000000000370. 27992391 10.1097/AUD.0000000000000370 PMC5313078

[bibr2-2331216519872601] Davis R. Kozel P. Erway L. (2003) Genetic influences in individual susceptibility to noise: A review. Noise and Health 5(20): 19. 14558889

[bibr3-2331216519872601] Fulbright, A. N. (2016). *Normal hearing or hidden hearing loss? What functional hearing tests, noise exposure history and ABR wave-I amplitude reveal* (PhD thesis). University of Florida, Gainesville, FL.

[bibr4-2331216519872601] Gallun, F. J., Diedesch, A. C., Kubli, L. R., Walden, T. C., Folmer, R. L., Lewis, M. S.,… Leek, M. R. (2012). Performance on tests of central auditory processing by individuals exposed to high-intensity blasts (Technical Report). Walter Reed National Military Medical Center, Bethesda, MD. doi: 10.1682/JRRD.2012.03.0038. 10.1682/jrrd.2012.03.0038 23341276

[bibr5-2331216519872601] Gatehouse S. Noble W. (2004) The speech, spatial and qualities of hearing scale (ssq). International Journal of Audiology 43(2): 85–99. doi: 10.1080/14992020400050014. 15035561 10.1080/14992020400050014 PMC5593096

[bibr6-2331216519872601] Gordon J. Griest S. Thielman E. Carlson K. Helt W. Lewis M. Henry J. (2017) Audiologic characteristics in a sample of recently-separated military veterans: The noise outcomes in servicemembers epidemiology study (noise study). Hearing Research 349: 21–30. doi: 10.1016/j.heares.2016.11.014. 27913314 10.1016/j.heares.2016.11.014

[bibr7-2331216519872601] Grinn S. K. Wiseman K. B. Baker J. A. Le Prell C. G. (2017) Hidden hearing loss? No effect of common recreational noise exposure on cochlear nerve response amplitude in humans. Frontiers in Neuroscience 11: 465 . doi: 10.3389/fnins.2017.00465. 28919848 10.3389/fnins.2017.00465 PMC5585187

[bibr8-2331216519872601] Grose J. H. Buss E. Hall J. W. III (2017) Loud music exposure and cochlear synaptopathy in young adults: Isolated auditory brainstem response effects but no perceptual consequences. Trends in Hearing 21: 2331216517737417 . doi: 10.1177/2331216517737417. 29105620 10.1177/2331216517737417 PMC5676494

[bibr9-2331216519872601] Guest H. Dewey R. S. Plack C. J. Couth S. Prendergast G. Bakay W. Hall D. A. (2018a) The noise exposure structured interview (NESI): An instrument for the comprehensive estimation of lifetime noise exposure. Trends in Hearing 22: 2331216518803213 . doi: 10.1177/2331216518803213. 30295145 10.1177/2331216518803213 PMC6176535

[bibr10-2331216519872601] Guest H. Munro K. J. Prendergast G. Millman R. E. Plack C. J. (2018b) Impaired speech perception in noise with a normal audiogram: No evidence for cochlear synaptopathy and no relation to lifetime noise exposure. Hearing Research 364: 142–151. doi: 10.1016/j.heares.2018.03.008. 29680183 10.1016/j.heares.2018.03.008 PMC5993872

[bibr11-2331216519872601] Henry J. A. Griest S. Zaugg T. L. Thielman E. Kaelin C. Galvez G. Carlson K. F. (2015) Tinnitus and hearing survey: A screening tool to differentiate bothersome tinnitus from hearing difficulties. American Journal of Audiology 24(1): 66–77. doi: 10.1044/2014_AJA-14-0042. 25551458 10.1044/2014_AJA-14-0042 PMC4689225

[bibr12-2331216519872601] Johnson T. A. Cooper S. Stamper G. C. Chertoff M. (2017) Noise exposure questionnaire: A tool for quantifying annual noise exposure. Journal of the American Academy of Audiology 28(1): 14–35. doi: 10.3766/jaaa.15070. 28054909 10.3766/jaaa.15070 PMC5304605

[bibr13-2331216519872601] Kujawa S. Liberman M. (2009) Adding insult to injury: Cochlear nerve degeneration after “temporary” noise-induced hearing loss. The Journal of Neuroscience 29(45): 14077–14085. doi: 10.1523/JNEUROSCI.2845-09.2009. 19906956 10.1523/JNEUROSCI.2845-09.2009 PMC2812055

[bibr14-2331216519872601] Le Prell C. G. Siburt H. W. Lobarinas E. Griffiths S. K. Spankovich C. (2018) No reliable association between recreational noise exposure and threshold sensitivity, distortion product otoacoustic emission amplitude, or word-in-noise performance in a college student population. Ear and Hearing 39(6): 1057–1074. doi:10.1097/AUD.0000000000000575. 29543608 10.1097/AUD.0000000000000575

[bibr15-2331216519872601] Levitt H. Rabiner L. (1967) Binaural release from masking for speech and gain in intelligibility. The Journal of the Acoustical Society of America 42: 601 . doi: 10.1121/1.1910629. 6073973 10.1121/1.1910629

[bibr16-2331216519872601] Liberman M. C. Epstein M. J. Cleveland S. S. Wang H. Maison S. F. (2016) Toward a differential diagnosis of hidden hearing loss in humans. PLoS One 11(9): e0162726 . doi: 10.1371/journal.pone.0162726. 27618300 10.1371/journal.pone.0162726 PMC5019483

[bibr17-2331216519872601] Littlefield P. D. Brungart D. (2019) Long-term sensorineural hearing loss in patients with blast-induced tympanic membrane perforations. Ear and Hearing. . doi:10.1097/AUD.0000000000000751. 10.1097/AUD.0000000000000751 31884502

[bibr18-2331216519872601] Miller J. D. (1974) Effects of noise on people. The Journal of the Acoustical Society of America 56(3): 729–764. doi: 10.1121/1.1903322. 4418709 10.1121/1.1903322

[bibr19-2331216519872601] National Institute for Occupational Safety and Health. (1998). Criteria for a recommended standard: Occupational noise exposure. Pub. no. 98-126. U.S. Department of Health and Human Services, Washington, DC. doi: 10.1121/1.4778162.

[bibr20-2331216519872601] Occupational Safety and Health Administration (1983) Occupational noise exposure: Hearing conservation amendment, final rule. Federal Register 48: 9738–9785.

[bibr21-2331216519872601] Saunders G. H. Frederick M. T. Arnold M. Silverman S. Chisolm T. H. Myers P. (2015) Auditory difficulties in blast-exposed veterans with clinically normal hearing. Journal of Rehabilitation Research & Development 52(3): 343–360. doi: 10.1682/JRRD.2014.11.0275. 26237266 10.1682/JRRD.2014.11.0275

[bibr22-2331216519872601] Sliwinska-Kowalska M. Pawelczyk M. (2013) Contribution of genetic factors to noise-induced hearing loss: A human studies review. Mutation Research/Reviews in Mutation Research 752(1): 61–65. doi: 10.1016/j.mrrev.2012.11.001. 10.1016/j.mrrev.2012.11.001 23207014

[bibr23-2331216519872601] Wightman F. Kistler D. (1992) The dominant role of low-frequency interaural time differences in sound localization. Journal of the Acoustical Society of America 91: 1648–1660. doi: 10.1121/1.402445. 1564201 10.1121/1.402445

